# Impact of Thioamide Derivative Composite Preservation System on Vulcanization of Natural Rubber

**DOI:** 10.3390/polym18040467

**Published:** 2026-02-12

**Authors:** Yuhang Hong, Liguang Zhao, Yazhong Song, Honghai Huang, Jianwei Li, Tuo Dai, Tao Zhao, Minmin Chen, Rentong Yu, Haoran Geng, Hongxing Gui, Jianhe Liao

**Affiliations:** 1 School of Materials Science and Engineering, Hainan University, Haikou 570228, China; 2 Hainan Natural Rubber Technology Innovation Center, Rubber Research Institute, Chinese Academy of Tropical Agricultural Sciences, Haikou 571101, China; 3 Sanya Quality Inspection and Testing Service Center, Sanya 572099, China

**Keywords:** natural rubber, preservation system, vulcanization, physical and mechanical properties

## Abstract

The thioacetamide derivative (TD)-composite preservation system (TDCPS) exhibits superior preservation effects on natural rubber (NR) latex and significantly enhances its vulcanization efficiency and mechanical properties. This study assessed TDCPS for NR, with a particular focus on its effects in promoting vulcanization. The TD containing both pyridine and thioamide groups was evaluated against other additives, namely thione accelerator ETU, pyridine 3-HP, and thioacetamide TAA. The results indicated that TD significantly reduced vulcanization time and enhanced efficiency, surpassing the moderate effects of ETU and 3-HP, as well as the minimal activity of TAA. Furthermore, TD and 3-HP demonstrated a synergistic effect in enhancing the properties of vulcanized NR, including elongation stress, tensile strength, tear resistance, and hardness, with TD achieving more rapid and complete vulcanization at higher dosages. Both TD and 3-HP increased the energy storage modulus of raw NR, thereby enhancing rigidity, while maintaining low loss factor values. The superior performance of TD is attributed to the synergistic interaction of its pyridine and thioamide groups, which optimize vulcanization kinetics and mechanical integrity. These findings underscore TD’s potential as an efficient vulcanization promoter for NR.

## 1. Introduction

In the current industrial context, the rubber sector holds a crucial position within the economy. Natural rubber (NR) products, renowned for their exceptional elasticity and durability, are extensively utilized across critical sectors such as automotive, construction, medical, and aviation [1]. However, NR latex is inherently prone to microbial degradation and deterioration, necessitating effective preservation strategies [2]. Traditionally, ammonia has been employed for this purpose; however, its high volatility and irritating properties pose significant environmental and safety challenges to its use [3,4]. In response to this challenge, the industry has developed ammonia-free preservatives, such as hexahydroxyethyl homotriazine, benzisothiazolinone, and bismorpholine methane, among others [5,6,7]. However, factors such as the preservation effect, toxicity of preservatives, and production costs hinder the large-scale dissemination and application of these preservatives. Among these, the thioamide derivative composite preservative system (TDCPS) emerges as a promising alternative, exhibiting significant advantages in terms of safety, low toxicity, consistent quality, and cost-effectiveness [8]. A key feature of TDCPS is its ability to significantly enhance the quality of NR latex while improving its physical and mechanical performance [9]. The core component of the system is a thioamide derivative (TD), which is complemented by a minimal quantity of auxiliary additives. Analytical studies have revealed that TD is uniquely responsible for accelerating and enhancing vulcanization within TDCPS. This functionality is attributed to the synergistic interplay of thioamide and pyridine functional groups in its molecular structure. These groups not only facilitate efficient cross-linking but also optimize the kinetics and extent of vulcanization, ultimately contributing to the production of high-performance rubber materials with enhanced mechanical properties [10,11,12,13,14].

NR, a primary raw material for rubber products, has long been the cornerstone of the rubber industry owing to its superior mechanical properties and environmental adaptability. Nevertheless, unprocessed NR exhibits certain constraints, notably low hardness and suboptimal abrasion resistance, restricting its use in high-performance applications [14]. Vulcanization, the process of creating a three-dimensional mesh structure between linear macromolecular chains, enhances these properties. This is achieved by introducing sulfur or other vulcanizing agents to the rubber, initiating a chemical reaction at a specified temperature. After vulcanization, rubber undergoes significant enhancements in its physical and mechanical attributes, rendering it suitable for high-performance applications [15,16]. Within this process, the role of the vulcanization accelerator is pivotal [17]. Vulcanization accelerators expedite the vulcanization process, lower the required temperature, augment the mechanical characteristics of the vulcanized rubber, and decrease sulfur usage, thereby reducing costs. Additionally, they bolster the dynamic fatigue performance, abrasion resistance, and aging resilience of the vulcanized rubber. Consequently, judicious selection of vulcanization accelerators is paramount to elevating the quality of rubber products [18].

Vulcanization accelerators are chemicals that expedite the vulcanization reaction, thereby enhancing the physical attributes of vulcanized rubber. These accelerators not only reduce the vulcanization duration and lower the vulcanization temperature but also augment the aging and abrasion resistance of the resultant rubber. In the rubber industry, judicious selection and utilization of a vulcanization accelerator is paramount to elevating product quality. Currently, prevalent vulcanization accelerators in the market encompass hyposulfonamide, thiazole, and thiuram, each of which exhibits distinct characteristics and finds application in specific scenarios, offering unique advantages [19]. For instance, hyposulfonamide vulcanization accelerators possess commendable delayed-vulcanization properties, making them apt for an array of rubber products [20], whereas thiazole accelerators are acclaimed for their rapid vulcanization capabilities, rendering them suitable for high-speed vulcanization processes [21]. Nevertheless, these conventional accelerators are not devoid of shortcomings; they may contain potentially hazardous substances and necessitate stringent operational conditions. With the expanding application of rubber products in extreme environments, there emerges an escalating demand for vulcanization accelerators capable of functioning proficiently under such challenging conditions [22].

Previous studies have established that the enhancement of the mechanical properties of NR-vulcanized films via composite preservation systems is primarily governed by the concentration of thioamide derivatives (TD). Building on this foundation, this study aimed to identify the specific chemical groups within TD responsible for its vulcanization-promoting effects. To achieve this, four key compounds—thioacetamide (TAA), thioketone accelerators (ETU), pyridine compounds, and TD—were selected as model additives. Their influence on the vulcanization behavior of NR blends and the resulting physical and mechanical properties of the vulcanized rubbers were systematically evaluated [23,24,25]. The experimental approach involved incorporating these four substances into fresh NR latex through a wet mixing process, followed by the production of raw rubber samples. The effects of each additive on the quality of raw rubber and the mechanical performance of the vulcanized products were characterized. This study specifically targeted the identification of key functional groups in TD that drive vulcanization enhancement and elucidated the underlying reaction mechanisms.

## 2. Experimental

### 2.1. Materials

NR latex was sourced from the Experimental Farm of the Chinese Academy of Tropical Agricultural Sciences in Hainan Province, China. Industrial-grade additives including TD, TAA, ETU, and 3-Hp (Table 1) were procured from Shandong Yusuo Chemical Technology Co. (Shandong, China). TDCPS was prepared in the laboratory. Analytical grade tetrahydrofuran and ammonia (25% wt) were supplied by Guangdong Xilong Chemical Co., Ltd (Shantou, China). Additional materials commonly used in the rubber industry, including ammonia, acetic acid, stearic acid, zinc oxide (ZnO), sulfur, and accelerator MBT(2-Mercaptobenzothiazole), were employed in the experiments.

### 2.2. Methods

#### 2.2.1. Preparation of NR Raw Rubber Samples

**Figure 1:**
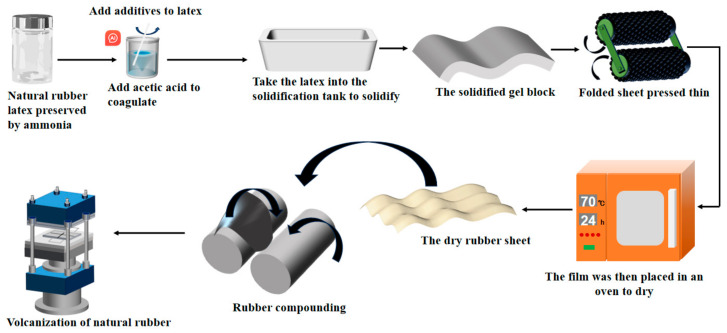
Process flow diagram for the preparation of both raw and vulcanized rubber utilizing natural latex blends.

A certain amount of fresh latex was collected from the rubber plantation and preserved using 0.1% ammonia. Subsequently, according to the formula in Table 2, the appropriate additives were added, mixed well, and left for 24 h. The appropriate amount of acid required to coagulate fresh latex was determined using the formula for calculating the amount of acid required for coagulation. After one day of standing, it was creased, dehydrated, and dried completely under hot air at 70 °C to prepare raw latex samples (Figure 1).

#### 2.2.2. Sample Preparation of Rubber Mixing and Vulcanization

The sample preparation adheres to the mixing procedure as stipulated in GB/T 6038-2006 [26]. The formula is as follows: Natural raw rubber 100 g, Stearic acid 0.5 g, Zinc oxide 6.09 g, Accelerator 0.5 g, Sulfur 3.5 g. Natural raw rubber was mixed using a two-roll mill (Jiangsu Mingzhu Testing Machinery Co., Ltd., Yangzhou, China) to obtain the rubber mixture. The vulcanization characteristics of this mixture were then examined using a rotorless vulcanizer (Jiangsu Tianyuan Testing Equipment Co., Ltd., Yangzhou, China) at a set temperature of 143 °C for 60 min. Subsequently, the mixed rubber was vulcanized using a plate vulcanizing machine (Jiangsu Tianyuan Testing Equipment Co., Ltd., Yangzhou, China), maintained at a temperature of 143 °C. The vulcanization process was carried out for a positive vulcanization time of t90 + 5 min, resulting in the formation of a vulcanized film.

#### 2.2.3. Preparation of Test Sample for Rubber Mixing

A pure rubber formulation was employed to produce 3.9 kg of compounded NR, which was subsequently divided into 13 equal 300 g portions. Subsequently, the additives were incorporated as per the specifications detailed in Table 3, ensuring thorough mixing. After allowing the mixture to rest for 12 h, its vulcanization characteristics were examined. Subsequently, a vulcanized rubber film was fabricated to assess its physical and mechanical properties.

#### 2.2.4. Testing of Raw Rubber

The Plasticity Retention Index (PRI) and Plasticity Initial Value (P0) were measured using a rapid plastometer (High-Speed Rail Testing Instruments (Dongguan) Co., Ltd., China, Dongguan) in accordance with GB/T 3510-2006 [27] and GB/T 3517-2014 [28], respectively. Mooney Viscosity [M_L_ (1 + 4) at 100 °C] was determined using a Mooney viscometer (Beijing Chuangcheng Zhijia Technology Co., Ltd., Beijing, China) following the GB/T 1232.1-2016 [29] standard. The nitrogen content was quantified using a Kjeldahl nitrogen analyzer (Shandong Holder Electronic Technology Co., Ltd., Shandong, China), as prescribed by GB/T 8088-2008 [30]. Fourier Transform Infrared Spectroscopy (FTIR) was performed using a TENSOR 27 spectrometer (Bruker Optics Inc., Billerica, MA, USA) in the range of 4000–370 cm^−1^ with a resolution of 4 cm^−1^. Thermogravimetric Analysis (TGA) was conducted using an STA449 analyzer (Netzsch-Geratebau GmbH, Selb, Germany); samples (10 mg) were heated from 25 °C to 600 °C at 10 K/min under a nitrogen atmosphere. The molecular weight and distribution were analyzed by Gel Permeation Chromatography (GPC) at 30 °C after dissolving the samples in tetrahydrofuran (THF). The dynamic viscoelastic properties (storage modulus G’ and loss factor tan δ) were analyzed using a Rubber Process Analyzer (RPA 2000, Alpha Technologies, Hudson, OH, USA) to evaluate the network structure. The glass transition temperature (T_g_) was determined using a differential scanning calorimeter (DSC, PerkinElmer Enterprise Management (Shanghai) Co., Ltd., Shanghai, China) in accordance with GB/T 29611-2013 [31].

#### 2.2.5. Determination of Mixing and Vulcanizing Rubber

The vulcanization speed of the rubber mix was determined using an MD-3000A rotorless vulcanometer (High-Speed Rail Testing Instruments (Dongguan) Co., Ltd., Dongguan, China). Subsequently, the vulcanization characteristics of the mix were examined at 143 °C for 40 min using the same instrument. The tensile strength, elongation at break, and modulus at fixed elongation of the vulcanized film were measured using an electronic universal testing machine (High-Speed Rail Testing Instruments (Dongguan) Co., Ltd., Dongguan, China). Tear strength was determined by using the same machine. Specimens for the tensile strength and right-angled tests were prepared in accordance with ISO 527 [32]. The tests were conducted using an electronic universal testing machine. The tensile strength, elongation at break, and constant elongation stress were evaluated according to the guidelines of GB/T 528-2009 [33], while the tearing strength was assessed based on GB/T 529-2008 [34]. Dumbbell and right-angle specimens required for these tests were prepared according to ISO 527. Finally, the stress–strain curves and relevant values of the samples were obtained using a universal electronic testing machine.

## 3. Results and Discussion

### 3.1. Impact of the Four Additives on the Vulcanization Characteristics and Physical and Mechanical Properties of the Compound Rubber

#### 3.1.1. Influence of the Four Additives on the Vulcanization Characteristics of the Compound Rubber

**Figure 2:**
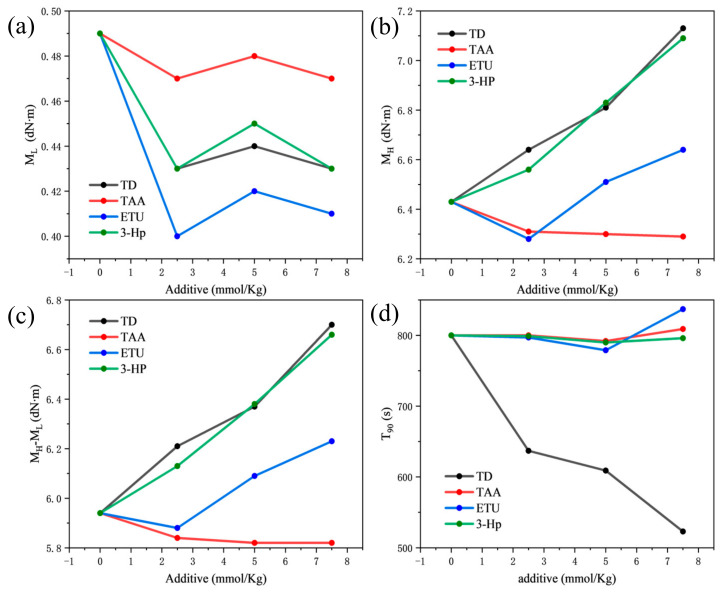
Effect of the four additives with 5 mmol/kg on (**a**) M_L_, (**b**) M_H_, (**c**) M_H_-M_L_, and (**d**) T90 of compound NR at 143 °C for 40 min. Lines connect data points for visual clarity.

**Figure 3:**
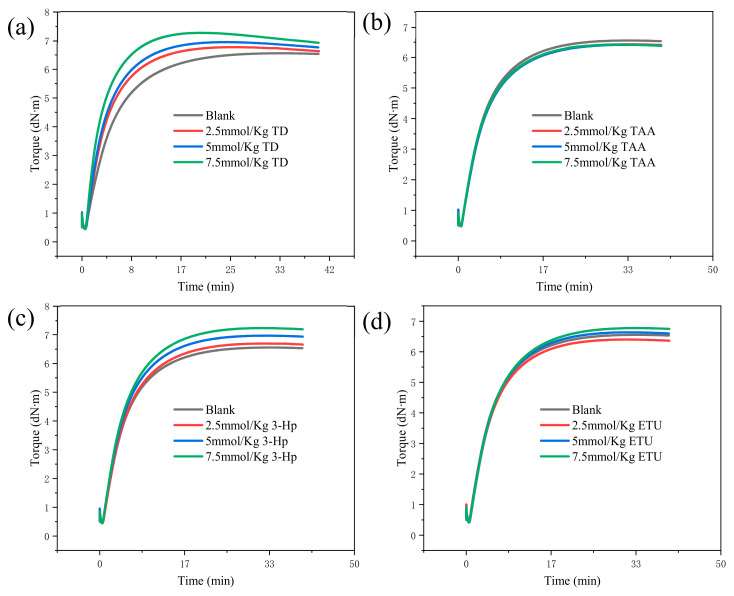
Vulcanization curves of compound NR with 5 mmol/Kg (**a**) TD, (**b**) TAA, (**c**) ETU, and (**d**) 3-Hp at 143 °C for 40 min.

Figure 2 and Figure 3 depict the trends in the vulcanization characteristics of pure rubber formulations as the four additives were modified. The minimum torque (M_L_) value serves as an indicator of the viscosity or fluidity of the compound rubber at the onset of vulcanization; a lower M_L_ value indicates superior processability. An increase in the maximum torque (M_H_) value results in an increased torque difference (M_H_-M_L_), which has direct implications for the hardness, elasticity, and other physical and mechanical attributes of the vulcanized rubber. This torque difference is a crucial metric for gauging the extent of hardening during vulcanization of the compound. A substantial torque difference typically signifies the commendable vulcanization responsiveness of the compound. Furthermore, vulcanization time provides insights into whether the additives accelerate or decelerate the vulcanization process.

Figure 2a illustrates that as the dosage of the four different additives increases, the M_L_ value initially declines, subsequently rises, and finally decreases again. However, the variations in M_L_ values were relatively small. This indicates that these four additives had a marginal effect on the ML value, suggesting that they maintain the processing fluidity of the rubber mix without significant alteration. When comparing the ML values with the same additive content, the order was TAA > 3-HP > TD > ETU. This suggests that under identical conditions, TAA exhibits the poorest processing performance, whereas ETU exhibits the best. Figure 2b,c reveal a consistent trend for M_H_ and the M_H_-M_L_; that is, an increase in the content of the four additives leads to a corresponding increase in M_H_ and M_H_-M_L_. Furthermore, at the same additive content, the lowest values of M_H_ and M_H_-M_L_ were observed for TAA, and the highest for TD. This demonstrates that TD has superior vulcanization responsiveness, significantly enhancing the properties of the material.

As illustrated in Figure 3, TD exhibited the most pronounced effect on enhancing the vulcanization curve, characterized by the swiftest rise in the degree of vulcanization and peak torque value. Concurrently, an increase in TD dosage elevates the torque value and markedly reduces the vulcanization time, although all exhibit vulcanization reversion. Subsequently, 3-Hp notably promoted vulcanization, as evidenced by the accelerated vulcanization speed and heightened torque value, with the reversion of vulcanization phenomenon being less pronounced than that with TD. Conversely, ETU exerts a comparatively weaker promoting effect on the vulcanization of NR and may even diminish the vulcanization speed at lower dosages. Finally, TAA did not enhance the vulcanization of the compound and, to a certain extent, reduced the degree of vulcanization. Among the four additives, only the vulcanization time of the TD compounds was curtailed with increasing TD dosage, thereby significantly expediting the vulcanization process, whereas the other three additives exerted a lesser impact on vulcanization time.

#### 3.1.2. The Impact of Four Additives on the Physical and Mechanical Properties of Vulcanized Rubber

**Figure 4:**
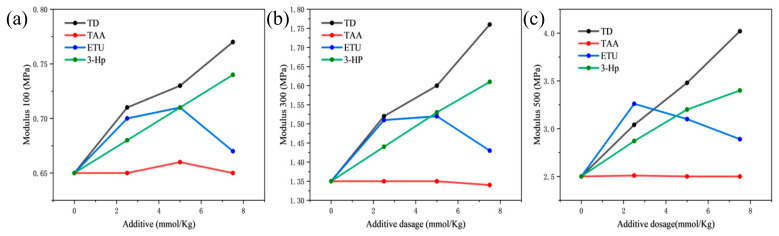
Effects of the four additives on (**a**) Modulus 100, (**b**) Modulus 300, and (**c**) Modulus 500 of NR vulcanizate. Lines connect data points for visual clarity.

The constant elongation stress of vulcanized rubber is intimately associated with its crosslink density. As illustrated in Figure 4, an increase in additive dosage, except for TAA, results in varying degrees of enhancement in the constant elongation stress of vulcanized NR. Specifically, the samples of vulcanized rubber film treated with TD and 3-Hp exhibited a continuous rise in constant elongation stress as the additive dosage increased, demonstrating similar patterns of change. However, the rate and extent of improvement were notably more pronounced in the TD group. In contrast, the constant elongation stress for the vulcanized rubber in the ETU group showed an initial increase followed by a decrease, whereas the samples with TAA remained largely unchanged. Furthermore, it can be deduced that the enhancement in elongation provided by TD is primarily attributed to the pyridine group, with other groups offering significant auxiliary synergistic contributions.

**Figure 5:**
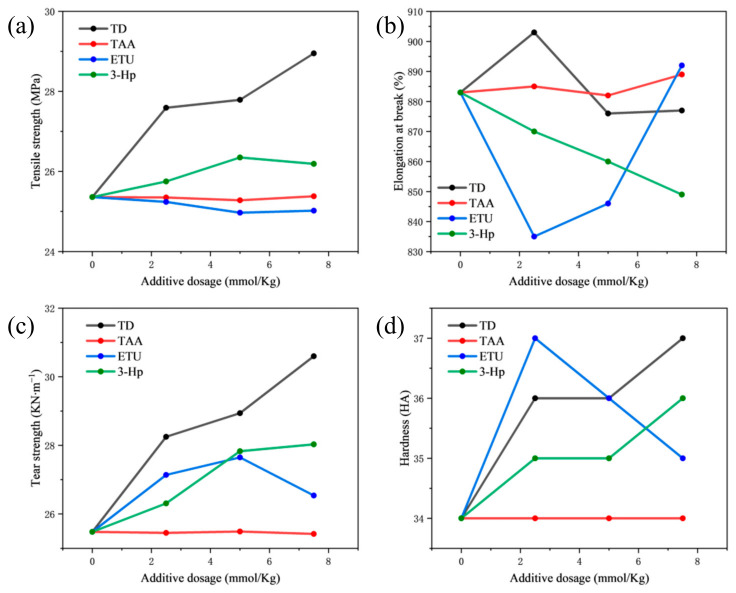
Effects of the four additives on (**a**) tensile strength, (**b**) elongation at break, (**c**) tear strength, and (**d**) hardness of NR vulcanizate. Lines connect data points for visual clarity.

The physical and mechanical properties primarily encompass the tensile strength, elongation at break, tear strength, and hardness. As depicted in Figure 5, the tensile strength of vulcanized NR experienced a marked increase significantly with increasing TD dosage. However, at lower dosages, 3-Hp also enhanced the tensile strength, but this effect plateaued when the dosage exceeded 5 mmol/Kg. Conversely, TAA and ETU exerted minimal influence on the tensile strength. The elongation at break for the vulcanized rubber demonstrated an initial increase, followed by a decrease as the TD dosage increased. The impact of TAA on these properties was marginal. An increase of 3-Hp dosage led to a consistent reduction in the elongation at break, whereas an increasing ETU dosage exhibited an initial decrease, followed by an increase. The tear strength of vulcanized NR consistently improved with increasing TD dosage, showing the most significant increase. The effect of 3-Hp dosage stabilized after a certain point, whereas an increased ETU dosage initially increased and then decreased. The influence of TAA on tear strength was notably less pronounced. The hardness of the vulcanized rubber augmented with both TD and 3-Hp dosages, yet the increase with ETU displayed a pattern of initial growth, followed by a decrease. The role of TAA in altering the hardness was relatively minor in this study. The analysis indicates that the enhanced physical and mechanical properties of the materials post-TD addition are predominantly attributed to the pyridine group within TD, with the thioamide group providing supplementary contributions.

#### 3.1.3. Result Analysis of Vulcanization Characteristics and Physical and Mechanical Properties

The variations in the physical and mechanical properties of the vulcanizates can be attributed to the differences in cross-link density and network structure induced by the additives. In this study, the torque difference M_H_-M_L_ derived from the vulcanization curves serves as an indirect indicator of the cross-link density.TD exhibited the highest M_H_-M_L_ value (Figure 2c), suggesting it formed the highest cross-link density among the samples. This high cross-link density correlates with the superior tensile strength and stress at fixed elongation observed in the TD vulcanizates. The synergistic action of the thioamide group (providing sulfur radicals) and the pyridine group (coordinating with zinc ions) likely facilitates a more efficient vulcanization network formation. In contrast, while 3-Hp also showed high torque values likely due to hydrogen bonding interactions, its lower elongation at break suggests a more rigid network structure. ETU resulted in a moderate torque difference, indicating a lower cross-link density compared to TD, which explains its moderate mechanical strength. TAA exhibited the lowest torque difference, confirming its poor activity and inability to form an effective cross-linked network, resulting in properties resembling unvulcanized rubber.

Compared to conventional vulcanization accelerators documented in the literature, TD presents a compelling combination of advantages. Its vulcanization efficiency, evidenced by the significantly reduced T_90_, surpasses that of typical thiazole-type accelerators like MBT [21], while approaching the activity of ultra-accelerators but with better processing safety. Mechanically, the tensile strength and modulus at fixed elongation achieved with TD are comparable to or exceed those reported for common sulfenamide accelerators like CBS [20]. Importantly, from a safety and environmental perspective, TD offers a distinct advantage over ethylene thiourea (ETU), which is a regulated substance due to toxicity concerns [22,24]. Therefore, TD emerges not only as an effective component of the preservation system but also as a high-performance and potentially safer alternative to several established vulcanization promoters.

### 3.2. Characteristics of NR Prepared by Wet Mixing with Four Additives

#### 3.2.1. Conventional Indicators of NR Raw Material

Fresh latex was processed into raw NR through wet mixing with four distinct additives. Mooney viscosity is a critical parameter for evaluating the processing fluidity of rubber, as it indicates the shear characteristics of rubber during the mixing process. As shown in Table 4, the raw rubber samples exhibited the highest Mooney viscosity upon the addition of ETU. Conversely, Mooney viscosity decreased with the incorporation of TAA, 3-HP, and TD. Plasticity provides insight into the malleability of rubber, and the retention of plasticity is a key index for assessing the resistance of rubber to thermo-oxidative degradation. As shown in Table 4, the initial plasticity value of the raw rubber displayed minimal variations upon the addition of the four additives. Notably, 3-HP and TAA reduced plasticity slightly. However, all four additives reduced the plasticity retention of raw rubber. Among them, ETU and 3-HP caused a more pronounced decline, whereas TD and TAA caused a relatively lesser decrease, indicating that their structures may offer better protection against oxidative chain scission during heating Furthermore, alterations in nitrogen content could be attributed to either the inherent nitrogen in the additives or their potential to inhibit microbial degradation reactions, thereby preserving a higher protein content. Specifically, the elevated nitrogen content observed in the TD-enhanced raw rubber samples may be correlated with TD’s potent antimicrobial and anti-mold properties of TD.

#### 3.2.2. The Impact of Various Additives on the Vulcanization Properties of Raw Rubber

**Figure 6:**
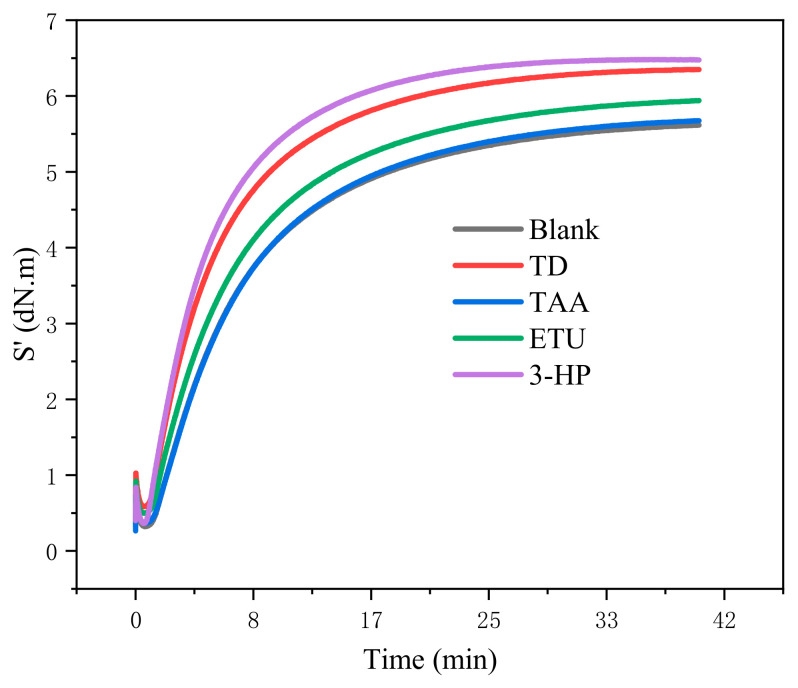
Vulcanization curves of compound NR with four additives at 143 °C for 40 min.

Figure 6 and Table 5 present the vulcanization characteristics of pure rubber formulations derived from fresh latex processed via wet mixing with four distinct additives. Contrary to the trend depicted in Figure 2a, all four additives enhanced the Maximum Latent Hardness of the compounds. Notably, ETU and TD exhibited the most significant enhancement in the ML of the compounds. A strong correlation was observed between the Maximum Hardness and the difference between the Maximum Hardness and Maximum Latent Hardness of the compounds. The compounds into which 3-HP was incorporated demonstrated superior M_H_ and M_H_-M_L_ values, followed by TD. Meanwhile, ETU had a comparatively minor enhancing effect on M_H_ and M_H_-M_L_ values, whereas TAA reduced these values. With respect to vulcanization time, the samples containing 3-HP displayed the shortest duration and the fastest rate of vulcanization across all stages, followed by TD. Both ETU and TAA expedited the vulcanization process, albeit with relatively weaker effects.

#### 3.2.3. Effects of Different Additives on the Physical and Mechanical Properties of Vulcanized Rubber

Table 6 presents the physical and mechanical properties of NR vulcanized from fresh latex using wet mixing with four additives. The quality of vulcanized rubber is determined by its physical and mechanical characteristics. Key metrics, such as modulus at fixed elongation, tensile strength, elongation at break, tear strength, and hardness, are detailed. At 100%, 300%, and 500% elongations, the samples containing TD and 3-Hp displayed elevated tensile stresses, with 3-Hp achieving the highest values at each modulus at fixed elongation, suggesting an enhanced load-bearing capacity. The modulus at fixed elongation for the TAA samples diminished. For tensile strength, TD and 3-Hp samples exhibited values of 25.90 MPa and 26.49 MPa, respectively, while TAA samples showed 18.47 MPa, lower than that of the blank control group. The elongation at break remained consistent across the additives, with ETU showing the highest value at 902%. The highest tear strength was recorded for 3-Hp at 27.9 kN∙m^−1^, followed by TD at 26.4 kN∙m^−1^. While the ETU samples showed robust tear strength, the TAA samples showed notably lower strength. For hardness, TD and 3-Hp samples were stiffer, whereas ETU and TAA showed less variation. In conjunction with the previously discussed vulcanization characteristics and properties, the causes of these occurrences were analyzed as follows: TD exhibited superior performance in enhancing the mechanical properties through its pyridine group promoting vulcanization [35]. Specifically, the pyridine group acts as a catalyst to significantly shorten the optimum cure time (T_90_), as shown in Table 5. TD creates cross-links in NR via covalent and hydrogen bonds, forming a network that increases the tensile strength. The TD improves the rubber properties by releasing reactive sulfur to form disulfide bonds, thereby increasing the crosslink density and interactions [36]. This enhances the tensile strength and hardness while maintaining elongation through a uniform network distribution [37]. The sulfur moiety strengthens the bonding by coordinating with the rubber double bond [38,39,40]. While 3-Hp’s hydroxyl group improves the tensile strength via hydrogen bonding, it causes brittleness and uneven crosslinking. TAA’s structural stability reduces vulcanization effectiveness. ETU and TD showed better network optimization than 3-Hp’s strength-brittleness trade-off and TAA’s inertness [41]. ETU accelerates vulcanization by facilitating the rapid formation of active sulfurating agents and forming polysulfide bonds. Although ETU cures faster than TD, its limited sulfur elongation restricts the tensile stress [42]. TAA exhibited the weakest mechanical properties, confirming its poor vulcanization and hardness owing to reduced crosslinking and network formation [43,44,45].

#### 3.2.4. Effects of Different Additives on the Relative Molecular Weight Size and Distribution of Raw NR

**Figure 7:**
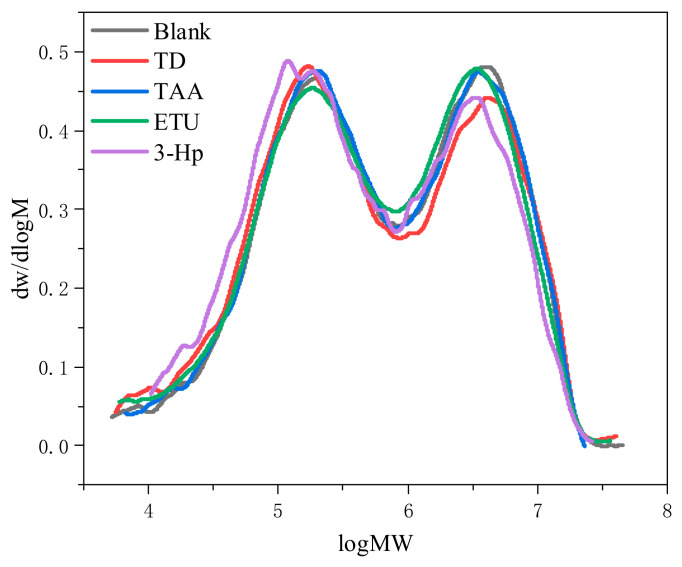
Molecular weight distribution of raw NR prepared with different additives.

The molecular weight significantly influences the processing performance of rubber materials and the mechanical properties of the final product. Figure 7 and Table 7 present the relative molecular mass distribution and molecular weight detection results of the soluble fraction of raw NR prepared by wet mixing with the four additives, respectively. As shown in Figure 7, the relative molecular mass distributions of the additive-treated raw NR were not notably different from those of the control group, exhibiting a consistent bimodal distribution. The samples with 3-Hp showed differences in the distribution plots, with higher low-molecular-weight and lower high-molecular-weight fractions. Conversely, the TD-added samples showed minimal variation in the low-molecular-weight distribution compared to the 3-Hp samples, but their high-molecular-weight fractions were similarly reduced. The weight-average molecular weights of the soluble fraction of raw NR with the four additives decreased compared to that of the control. TD showed an insignificant decline, whereas 3-HP displayed the most pronounced reduction. TAA led to a slight enhancement in the number-average molecular weight. However, these weights decreased with ETU and TD, with TD causing a greater decrease. The number-average molecular weight of 3-HP remained consistent with that of the control. The distribution coefficient increased due to TD but decreased with other additives, with 3-HP showing the sharpest decrease. These findings indicate that the addition of TD increased the number of smaller molecular weight components. Concurrently, the molecular weight of the larger components increased, broadening the distribution and amplifying the molecular weight disparity among the rubber chains. With 3-HP introduction, smaller-molecular-weight components increased, larger molecules decreased, and the molecular weight distribution became more tightly clustered. These observations are consistent with the results shown in Figure 7. It is important to note that GPC analysis characterizes only the soluble fraction (sol) of the rubber. While it does not directly depict the cross-linked network structure formed during vulcanization, the changes in the molecular weight of the raw rubber provide insight into how the additives influence the polymer chains during the initial processing stages. A distinct variation in the molecular weight distribution can influence the processing fluidity (Mooney viscosity) and partially affect the ultimate mechanical properties of the vulcanizates.

The 3. HP displayed the most substantial reduction. The incorporation of TAA marginally increased the number-average molecular weight. However, it diminished with the inclusion of both ETU and TD, with TD witnessing a sharper drop. Notably, the number-average molecular weights after 3-Hp addition aligned with those of the control group. The distribution coefficient increased upon TD addition but decreased with the other three additives, with 3-Hp causing the most pronounced reduction. After TD addition, the proportion of smaller-molecular-weight components increased, the molecular weight of the larger components increased, and the molecular weight distribution broadened. Conversely, after 3-Hp addition, there was a surge in smaller molecular weight components and a decrease in the count and molecular weight of larger molecules, leading to the tightest molecular weight distribution. These observations are consistent with the results shown in Figure 7.

#### 3.2.5. Effects of Different Additives on the Preparation of Infrared Spectrum of NR

**Figure 8:**
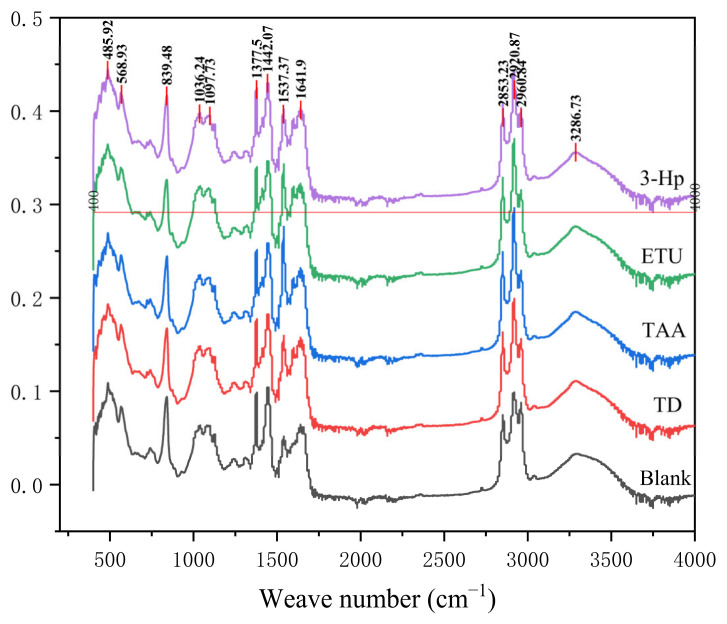
Infrared spectrum of raw NR prepared with different additives.

Figure 8 shows the infrared spectrum of NR derived from fresh latex prepared via wet mixing with four distinct additives. As shown in Figure 8, there are no noticeable shifts in the wave peaks of the infrared spectra of the five NR samples. Moreover, the intensities of these wave peaks did not exhibit significant changes. The characteristic peaks of all samples essentially fell within the same number of bands, and the overall infrared curve mappings were closely aligned, with minimal variance observed. The C=C double bond in the NR molecule corresponds to a wavenumber of 1645 cm^−1^, accompanied by a bending vibration peak at 840 cm^−1^. The -CH_3_ and -CH_2_ groups exhibit wave numbers of 2959 cm^−1^ and 2851 cm^−1^, respectively, with their bending vibration peaks manifesting near wave numbers of 1443 cm^−1^ and 1375 cm^−1^. Notably, the six profiles exhibited minor discrepancies in certain characteristic peaks, suggesting that the integration of the four molecules might not be optimal for the infrared profile. These slight variations in the characteristic peaks across the six profiles imply a marginal structural impact on NR after the addition of the four substances. Consequently, drawing definitive conclusions about their influence on the various properties of dried rubber films is challenging.

#### 3.2.6. Influence of Different Additives on the Thermal Stability of Raw NR

**Figure 9:**
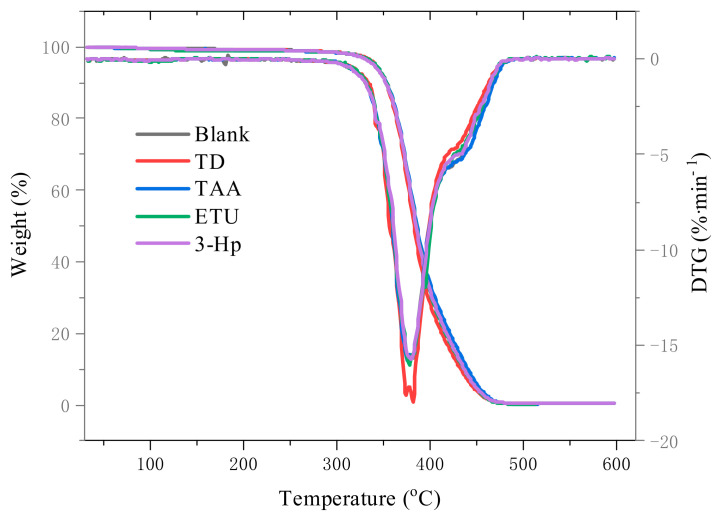
The Thermogravimetric and derivative thermogravimetric curves of raw NR prepared with different additives.

The Thermogravimetric (TG) and Derivative Thermogravimetric (DTG) profiles of raw NR prepared from fresh latex with the four additives and their characteristic temperatures are presented in Figure 9 and Table 8. The impact of the additives on NR’s thermal stability of NR was assessed using the initial decomposition temperature (T_0_). Samples with TD and 3-Hp exhibited marginally higher onset decomposition temperatures, suggesting enhanced initial thermal stability. The TAA and ETU samples exhibited decomposition temperatures comparable to those of the blank samples, indicating a minimal effect on the initial thermal stability. For T50, 3-Hp recorded the highest value at 386.28 °C, while TD displayed a lower T50%, indicating faster mid-period decomposition. Other additives minimally influenced the T50%. The peak decomposition temperature (T_p_) varied with the additives, with TD showing the highest value at 382.00 °C, followed by 3-HP, whereas TAA showed the lowest value. The final temperature (T_f_) for the TAA and ETU samples was marginally higher than that of the blanks, with minor differences between the 3-HP and control samples, whereas the TD was lower. While all additives elevated the initial decomposition temperature, TD showed inferior overall thermal stability, whereas the others demonstrated slightly enhanced stability compared to the controls. For TD, despite the pyridine group improving the initial stability, thioamide degradation and uneven crosslinking reduced the thermal stability later [46]. With 3-HP, hydrogen bonding and pyridine group synergy enhanced the molecular chain stability [47]. TAA’s low reactivity provided minimal thermal stability improvement. ETU’s early vulcanization due to rubber double bonds forms sulfur bonds, slightly improving the thermal stability [48,49].

#### 3.2.7. Influence of Different Additives on RPA Data of Raw NR Preparation

**Figure 10:**
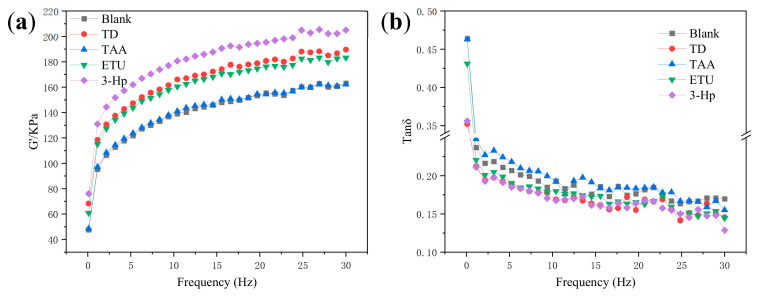
(**a**) G’ and (**b**) tanδ curves of raw NR with the four additives.

The analysis of G’ (energy storage modulus) and tan δ (loss factor) curves is crucial for understanding the dynamic mechanical properties of rubber materials. These curves provide insights into the elastic behavior and energy dissipation characteristics at varying frequencies and temperatures. As shown in Figure 10a,b, the incorporation of TD, 3-HP, and ETU enhanced the G’ values across the test range. 3-HP showed the strongest effect, followed by TD, suggesting increased rubber rigidity due to the synergistic effect of TD and 3-HP, which promotes cross-linking and strengthens the network structure. Changes in the tan δ curve indicate shifts in viscoelasticity. A peak shift to a higher temperature or frequency indicates a restricted segmental motion of the polymer chains, indicating reduced flexibility and enhanced thermal stability. TAA minimally influences the G’ and tanδ of raw rubber samples. ETU slightly elevated G’ and reduced tanδ, which was similar to the TD effects. Samples with 3-HP showed the highest G’ curve and lowest tanδ. The causes are:

- 3-HP: (1) Hydrogen bonding strengthens molecular chain interactions through reversible bonds between hydroxyl groups (-OH) and rubber chains, increasing crosslinking density and improving rigidity [41]. (2) Pyridine groups coordinate with Zn^2+^ (Zn(C5H5N)^2+^), optimizing the crosslinked network homogeneity and reducing energy dissipation [40]. (3) Hydrogen bonding and ligand interactions form a stable crosslinking network, enhancing elastic storage and suppressing viscous dissipation.
- Thioamide Derivatives (TD): (1) Thioamide enables cross-linking through sulfur radicals from R-C=S groups, accelerating sulfur cross-linking [38,39]. (2) Pyridine-Zn^2+^ coordination increases network rigidity, although uneven crosslinking leads to incomplete chain movement restriction. (3) Thioamides may cause partial chain breaks during vulcanization, thereby increasing energy dissipation.
- The properties of ethylene thiourea (ETU) are as follows: (1) Rapid crosslinking forms short sulfur bonds (-S- or -S-S-), creating a brittle network. (2) Short sulfur bonds break and reconnect during deformation, causing viscous dissipation [42]. (3) High crosslink density trades off with network uniformity, limiting the optimization of the elastic response.
- Thioacetamide (TAA): (1) Limited crosslinking: The thioamide group in TAA has low reactivity, preventing effective vulcanization and having a negligible impact on the crosslinked network. (2) Unrestricted Molecular Chain Movement: No cross-linking points are formed, maintaining the viscoelastic properties of the rubber (G’ ≈ blank, tanδ ≈ blank) [43,44].

**Figure 11:**
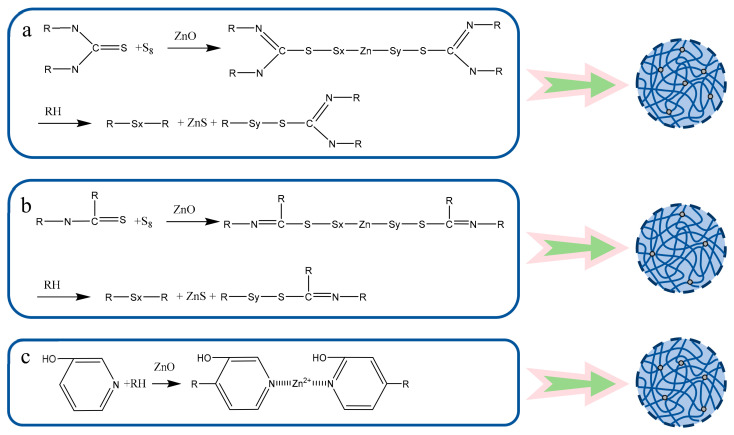
Promoting mechanism of (**a**) TD, (**b**) ETU and (**c**) 3-Hp on rubber vulcanization.

TD promotes a uniformly high crosslink density by slowly releasing reactive sulfur and forming long sulfur bridges. This process fully extends the sulfur bonds, enhancing network stability with only a slight increase in the elongation at break [50]. In contrast, ETU accelerates vulcanization, generating dense short sulfur bonds that form a dense and flexible network. This resulted in an 8.9% increase in the tear strength, although the magnitude of tensile enhancement was lower than that of the TD direction [42,51]. 3-Hp selective crosslinking leads to local overdensification and improves the tensile strength. However, hydrogen-bond-induced brittleness results in a 14.0% decrease in tear strength [52]. TAA, owing to its low reactivity, had no significant effect on the tensile strength. However, hydrogen bonding-induced brittleness results in a 14.0% decrease in tear strength [42,51]. The crosslink density positively enhanced the strength and hardness of the material. However, the tearing performance is dependent on the sulfur-bridge length and network homogeneity. The flexible structure of ETU is superior to the inhomogeneous distribution of 3-Hp, whereas TD balances high strength with moderate elongation. The mechanisms by which the three additives (TD, ETU, and 3-Hp) promoted NR vulcanization are shown in Figure 11. As depicted in this figure, all three components facilitated the crosslinking of rubber molecules, thereby significantly enhancing the mechanical properties of the rubber.

## 4. Conclusions

This study aimed to examine the key chemical groups of thioamide derivative (TD) present in the composite preservation system to amplify the vulcanization properties of NR. The distinct impacts of various additives, namely TD, TAA, ETU, and 3-Hp, on the preparation of NR blends and vulcanized rubber were meticulously analyzed. The findings revealed that TD significantly reduced the vulcanization time and exhibited potent vulcanization-promoting capabilities. Conversely, TAA exerted virtually no beneficial influence on vulcanization, whereas ETU and 3-Hp exhibited varying degrees of vulcanization promotion. Notably, the TD samples demonstrated an accelerated vulcanization speed and a heightened degree of vulcanization at increased dosages. In contrast, TAA’s influence of TAA on the performance of vulcanized rubber was virtually imperceptible, and the effect of ETU was also relatively constrained.

The raw rubber produced by incorporating TD into fresh latex via wet mixing exhibited a reduced number-average molecular weight and a broader molecular weight distribution. Similarly, the ETU sample displayed a diminished weight-average molecular weight with a relatively narrow molecular weight distribution coefficient. The 3-Hp sample also presented a decreased weight average molecular weight, and its molecular weight distribution coefficient was notably the lowest among the samples. The infrared spectroscopy results revealed consistent characteristic peaks across the five samples, with no marked migration or intensity variations, suggesting a minimal impact of the additives on the primary chain structure of NR. Notably, the raw rubber samples treated with TD and 3-Hp exhibited an elevated energy storage modulus (G’), indicating increased rigidity of the rubber molecules. Concurrently, their loss factor (tanδ) curves diminished, with the 3-Hp samples showing particularly low tanδ values.

In summary, experimental data confirm that TD acts as a highly efficient promoter, significantly reducing the optimum cure time (T_90_) and increasing the torque difference (M_H_-M_L_), which indicates the formation of a higher cross-link density. When compared to conventional vulcanization accelerators, TD demonstrates a distinctive combination of rapid curing efficiency that exceeds typical thiazole accelerators, mechanical properties on par with established sulfenamide-based systems, and a more favorable environmental and safety profile relative to regulated substances such as ethylene thiourea ETU. Consequently, the incorporation of TD not only markedly accelerated the vulcanization process and enhanced the degree of vulcanization of NR but also significantly improved the physical and mechanical properties of the vulcanized NR. Furthermore, the mechanism suggests TDCPS could potentially apply to synthetic rubbers with unsaturated double bonds.

## Figures and Tables

**Table 1:** Chemical structure of four additives.

Category	TD	TAA	ETU	3-Hp
Chemical structure	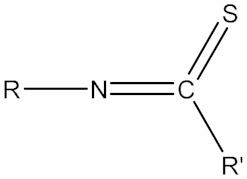	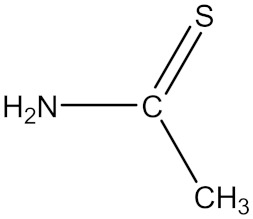	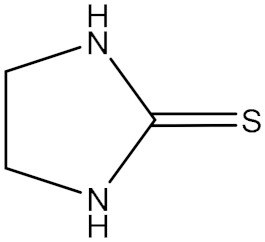	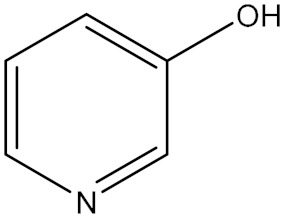

**Table 2:** NR latex additive composition and dosage.

Additives	Dosage/mmol.kg^−1^
Blank	-
TD	5
TAA	5
ETU	5
3-Hp	5

Note: The additives utilized were either aqueous solutions or colloidal suspensions, with the dosage indicating the optimal quantity of preservatives employed for the preservation of natural latex.

**Table 3:** Dosage of additives for compounded NR.

Additives	Dosage/mmol.kg^−1^
blank	-
TD	2.5, 5, 7.5
TAA	2.5, 5, 7.5
ETU	2.5, 5, 7.5
3-Hp	2.5, 5, 7.5

**Table 4:** Conventional indexes of raw NR prepared by mixing fresh latex with the four additives.

Additives	Mooney Viscosity	P_0_	PRI/%	Nitrogen Content/%
-	71.57	34.8	86.2	0.407
TD	68.74	35.2	82.61	0.478
TAA	70.20	33.7	83.14	0.466
ETU	73.58	34.4	77.21	0.399
3-Hp	69.62	32.1	73.13	0.419

**Table 5:** Effects of different additives on vulcanization characteristics of fresh latex prepared compound.

Additives	M_L_/dN∙m	M_H_/dN∙m	M_H_-M_L_/dN∙m	T_10_/s	T_50_/s	T_90_/s
-	0.42	5.62	5.2	130	350	1169
TD	0.69	6.35	5.66	91	226	826
TAA	0.49	5.47	4.98	142	367	1192
ETU	0.61	5.94	5.33	117	313	1097
3-Hp	0.47	6.47	6	80	208	774

**Table 6:** Effects of different additives on mechanical properties of vulcanized NR prepared from fresh latex.

Detection Index	Additives				
	-	TD	TAA	ETU	3-Hp
Tensile stress at 100% elongation/MPa	0.59	0.68	0.55	0.61	0.70
Tensile stress at 300% elongation/MPa	1.21	1.50	1.14	1.31	1.57
Tensile stress at 500% elongation/MPa	2.49	3.29	2.34	2.80	3.55
Tensile strength/MPa	19.43	25.90	18.47	23.09	26.49
Elongation at break/%	882.4	898.7	899.7	902.0	881.0
Tear strength/kN∙m^−1^	20.8	26.4	21.0	23.0	27.9
Hardness/HA	33.3	37.0	32.8	34.8	37.7

**Table 7:** Relative molecular weight size and distribution coefficient of raw rubber with different additives.

Additives	Mn/10^4^	Mw/10^4^	Distribution Coefficient
-	13.02	236.52	18.17
TD	11.35	235.96	20.79
TAA	14.14	232.58	16.45
ETU	12.48	221.26	17.72
3-Hp	13.02	195.54	15.02

**Table 8:** Characteristic degradation temperature of NR latex dry films incorporating various additives.

	T_0_/°C	T_50_/°C	T_p_/°C	T_f_/°C
Blank	353.62	385.35	377.31	415.17
TD	354.69	383.58	382.00	411.98
TAA	353.88	385.39	376.51	418.01
ETU	353.98	385.70	377.85	416.20
3-Hp	354.61	386.28	380.08	415.97

## Data Availability

The original contributions presented in this study are included in the article. Further inquiries can be directed to the corresponding author.

## Abbreviations

NR	Natural rubber
TD	Thioacetamide derivative
TAA	Thioacetic acid
ETU	Thioketone accelerators
3-Hp	3-Hydroxypyridine
Mv	Mooney viscosity
M_L_	The minimum torque
M_H_	The highest maximum torque
phr	Parts per hundred rubber

## References

[1] Loykulnant S., Kongkaew C., Chaikumpollert O., Sanguanthammarong P., Na Ubol P., Suchiva K. (2011). Study of chitosan and its derivatives as preservatives for field natural rubber. J. Appl. Polym. Sci..

[2] Luo Y., Yang C., Li Z., Liao S. (2024). A study on preservatives on the structure and properties of natural rubber latex. J. Rubber Res..

[3] Afreen S., Haque K.R., Huda M.K. (2013). Troubleshooting for the observed problems in processing latex concentrate from natural resource. IOP Conf. Ser. Earth Environ. Sci..

[4] Masia B., Yang M., Cozzani V. (2024). Risk assessment of ammonia fueled ships: Consequences on human health of ammonia releases from damaged fuel storage tanks. ACS Chem. Health Saf..

[5] Wang T., Gui H.X., Zhang W.F., Zhang K.X., Yu W.Q., Li Y.M., Zeng R.Z., Huang M.F. (2015). Novel non-ammonia preservative for concentrated natural rubber latex. J. Appl. Polym. Sci..

[6] Zhao L., Gui H., Yang G., Ding L., Song Y., Li J., Huang H., Zhao T., Liu W. (2023). Properties of ammonia-free concentrated NR latex preserved with N,N’-methylene-bis-morpholine. Rubber Chem. Technol..

[7] Rojruthai P., Rodgerd P., Ho C.-C., Sakdapipanich J. (2021). The use of 1,2-benzisothiazolin-3-one (BIT) in preparation of low-ammonia and zinc-free natural rubber latex concentrate. J. Rubber Res..

[8] Zhao L., Xing P., Zhao L., Yang Q., Song Y., Ding L., Zhao T., Wang Y., Xin Z., Gui H. (2025). Optimization Study of a High-Efficiency Preservative for Ammonia-Free Concentrated Natural Rubber Latex. Polymes.

[9] Zhao L.G., Ding L., Zhao L.Y. (2024). Preservative effect of thione derivatives LS on natural rubber latex. Chin. J. Trop. Crops.

[10] Hu R., Zhao S., Chen F., Shangguan Y., Zheng Q. (2022). Effect of sacrificial bond on molecular dynamics and rheological behavior of hybrid butadiene-styrene-vinylpyridine rubber vulcanizates with reversible sacrificial network. J. Polym. Sci..

[11] Kurien M., Susamma A.P., Kuriakose A.P. (2004). Amidino thiourea as a secondary accelerator in the sulphur vulcanization of natural rubber containing fillers. Prog. Rubber Plast. Recycl. Technol..

[12] Yang S.Y., Jia Z.X., Liu L., Fu W.W., Jia D.M., Luo Y.F. (2014). Insight into vulcanization mechanism of novel binary accelerators for natural rubber. Chin. J. Polym. Sci..

[13] Tang Z., Huang J., Guo B., Zhang L., Liu F. (2016). Bioinspired engineering of sacrificial metal-ligand bonds into elastomers with supramechanical performance and adaptive recovery. Macromolecules.

[14] Whba R., Su’ait M.S., Whba F., Sahinbay S., Altin S., Ahmad A. (2024). Intrinsic challenges and strategic approaches for enhancing the potential of natural rubber and its derivatives: A review. Int. J. Biol. Macromol..

[15] Tayeb K.B., Eliard C., Vezin H., Gabrielle B., Delebecq E., Gomez E. (2022). In situ EPR investigation of sulfur vulcanization mechanism and ageing process. Polym. Degrad. Stab..

[16] Akahori Y., Kawahara S. (2023). Effect of water on the accelerated sulfur vulcanization of natural rubber. Polym. Test..

[17] Chen M., Zhou Y., Shen Z., Liu J., Gao R., Li X., Zhang L., Li F. (2023). A crosslinking kinetic model considering reversion effect with verification and its application in thick rubber vulcanization process. Polymer.

[18] Bhadra S., Mohan N., Krishna R.L., Nair S.S. (2022). Identification of glycerol as a novel accelerator for sulphur vulcanization of unsaturated rubbers. J. Elastom. Plast..

[19] Alam M.N., Kumar V., Potiyaraj P., Lee D.-J., Choi J. (2022). Synergistic activities of binary accelerators in presence of magnesium oxide as a cure activator in the vulcanization of natural rubber. J. Elastom. Plast..

[20] Charoeythornkhajhornchai P., Samthong C., Somwangthanaroj A. (2017). Influence of sulfenamide accelerators on cure kinetics and properties of natural rubber foam. J. Appl. Polym. Sci..

[21] Alam M.N., Mandal S.K., Debnath S.C. (2012). Effect of zinc dithiocarbamates and thiazole-based accelerators on the vulcanization of natural rubber. Rubber Chem. Technol..

[22] Fu Z., Jin H., Mao W. (2025). Thiuram vulcanization accelerators in human urine and their human exposure. Environ. Res..

[23] Berry K. (2014). The Quest for a Safer Accelerator for Polychloroprene Rubber. Ph.D. Thesis.

[24] Yarzabal I. (2020). Bis mercapto thiadiazole in polychloroprene for 1,3-ethylene thiourea (ETU) replacement. Chem. Mater..

[25] Zhang J., Huang S., Kong L., Sakdapipanich J., Zhang R., Xie Z., Wu J. (2024). Unveiling the hierarchical microstructure of prevulcanized natural rubber latex film and its impact on mechanical properties. Macromolecules.

[26] (2006). Rubber Test Mixes—Preparation, Mixing and Vulcanization—Equipment and Procedures.

[27] (2007). Rubber, Unvulcanized—Determination of Plasticity—Rapid Plastimeter Method.

[28] (2014). Rubber, Raw Natural—Determination of Plasticity Retention Index (PRI).

[29] (2016). Rubber, Unvulcanized—Determinations Using a Shearing-Disc Viscome-Ter—Part 1: Determination of Mooney Viscosity.

[30] (2008). Rubber, Raw Natural and Rubber Latex, Natural—Determination of Nitrogen Content.

[31] (2013). Rubber, Raw—Determination of the Glass Transition Temperature—Differential Scanning Calorimetry (DSC).

[32] (2019). Plastics—Determination of Tensile Properties—Part 1: General Principles.

[33] (2009). Rubber, Vulcanized or Thermoplastic—Determination of Tensile Stress-Strain Properties.

[34] (2008). Rubber, Vulcanized or Thermoplastic—Determination of Tear Strength (Trouser, Angle and Crescent Test Pieces).

[35] Alam M.N., Mandal S.K., Roy K., Debnath S.C. (2014). Synergism of novel thiuram disulfide and dibenzothiazyl disulfide in the vulcanization of natural rubber: Curing, mechanical and aging resistance properties. Int. J. Ind. Chem..

[36] Thungphotrakul N., Rattanaporn K., Jarastrakull P., Srinophakun P., Prapainainar P. (2025). Impact of Sulfur Vulcanization and Electron Beam Irradiation on the Properties of Antimicrobial Natural Rubber/Lignin Composite Films. ACS Omega.

[37] Jiang G., Wen Z., Yan S., Wang Z., Fang L., Tang H. (2025). Chelation of Zinc Ions by EDTA: A Novel Strategy for Stabilizing the Vulcanization Degree of Prevulcanized Natural Rubber Latex. J. Appl. Polym. Sci..

[38] Dey G.R. (2019). Effect of phenyl moiety on the formation of radicals and radical cations of thioamides in n-butyl chloride: A pulse radiolysis study. J. Chem. Sci..

[39] Datta R.N., Das P.K., Basu D.K. (1986). Studies on the reactions between thiocarbamyl sulfenamide and 2-(iminodithio) benzothiazole accelerator system in the early stage of vulcanization of NR. J. Appl. Polym. Sci..

[40] Zubenko A.D., Egorova B.V., Kalmykov S.N., Shepel N.E., Karnoukhova V.A., Fedyanin I.V., Fedorov Y.V., Fedorova O.A. (2019). Out-cage metal ion coordination by novel benzoazacrown bisamides with carboxyl, pyridyl and picolinate pendant arms. Tetrahedron.

[41] Chen Q., Huang W., Zhang L., Chen Y., Liu J. (2024). Impact of sacrificial hydrogen bonds on the structure and properties of rubber materials: Insights from all-atom molecular dynamics simulations. Langmuir.

[42] Naebpetch W., Nithi-Uthai N., Saetung A., Junhasavasdikul B., Kaewsakul W. (2017). Utilisation of zinc dimethacrylate as coagent in sulfur-peroxide dual vulcanisation with different sulfur systems for styrene-butadiene rubber compounds. J. Rubber Res..

[43] Datta R.N., Das P.K., Basu D.K. (1986). Effect of cyclohexyl thiophthalimide on NR vulcanization accelerated by thiocarbamyl sulfenamide-dibenzothiazyl disulfide system. Rubber Chem. Technol..

[44] Das P.K., Datta R.N., Basu D.K. (1987). Cure modification effected by 2-iminothiophthalimides in the vulcanization of NR accelerated by thiocarbamyl sulfenamides and dibenzothiazyl disulfide. Rubber Chem. Technol..

[45] Datta R.N., Basu D.K. (1986). Effect of zinc dithiocarbamates on NR vulcanization accelerated by thiocarbamyl sulfenamides and dibenzothiazyl disulfide. Rubber Chem. Technol..

[46] Wu R., Niu Z., Huang L., Xia Z., Feng Z., Qi Y., Dai Q., Cui L., He J., Bai C. (2022). Vanadium complexes bearing the bulky bis(imino)pyridine ligands: Good thermal stability toward ethylene polymerization. Eur. Polym. J..

[47] Tang C., Li X., Li Z., Hao J. (2017). Interfacial hydrogen bonds and their influence mechanism on increasing the thermal stability of nano-SiO2-modified meta-aramid fibres. Polymers.

[48] Yu K., Chen L., Tang Y., Ma A., Zhu W., Wang H., Tang X., Li Y., Li J. (2025). Enhanced thermostability of nattokinase by rational design of disulfide bond. Microb. Cell Fact..

[49] Tang F., Chen D., Yu B., Luo Y., Zheng P., Mao X., Yu J., He J. (2017). Improving the thermostability of Trichoderma reesei xylanase 2 by introducing disulfide bonds. Electron. J. Biotechnol..

[50] Wang Y., Liu H., Zheng T., Peng Z., Wang R., Yu H., Wang Q., Liao S., Liao L. (2023). Strain-induced crystallization behavior and tensile properties of natural rubber with different vulcanization bond types. Polym. Test..

[51] Surya I., Ismail H. (2016). Alkanolamide as a novel accelerator and vulcanising agent in carbon black-filled polychloroprene rubber compounds. Plast. Rubber Compos..

[52] Wang L., Fu W., Peng W. (2020). Enhanced strength and toughness of polyurethane rubber by introducing hydrogen bond sacrificial units at rubber-graphene interfaces. Polym. Compos..

